# Supplementary material for: Edible unclonable functions
Source: Nat Commun. 2020 Jan 16;11:328. doi: 10.1038/s41467-019-14066-5 (PMC6965141; doi:10.1038/s41467-019-14066-5)
Supplement: Supplementary file 1 — Supplementary Information [file 41467_2019_14066_MOESM1_ESM.pdf]

Supplementary Information

Edible unclonable functions

Leem et al.

Supplementary Figures

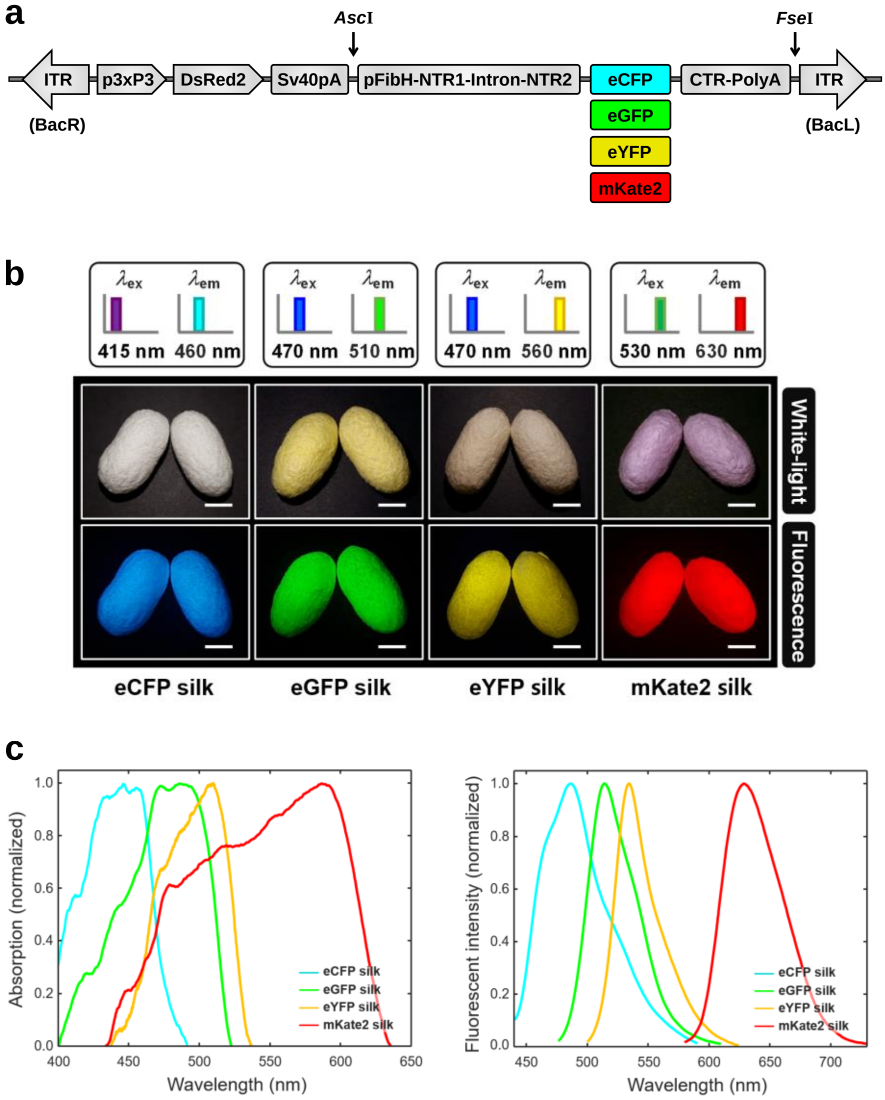

Supplementary Figure 1. Silkworm transgenesis and fluorescent silk. (**a**) Transformation vector of p3xP3-DsRed2-FibH-(eCFP, eGFP, eYFP, or mKate2) for fluorescent silk production in silkworm transgenesis. In the case of mKate2, eGFP is used instead of DsRed2. The nucleotide sequences of pFibH-NTR and CTR are derived from Genebank Accession No. AF226688. pFibH: fibroin heavy chain promoter domain (1124 bp), NTR1: N-terminal region 1 (142 bp), intron: first intron(871 bp), NTR2: N-terminal region 2 (417 bp), CTR: C-terminal region (179 bp), PolyA: poly(A) signal region (301 bp), eCFP: enhanced cyan fluorescent protein, eGFP: enhanced green fluorescent protein, eYFP: enhanced yellow fluorescent protein, mKate2: monomeric far-red fluorescent protein, DsRed2: red fluorescent protein (a mutant form of DsRed from *Discosoma sp.*), ITR: inverted repeat sequences of *piggyBac* arms, 3xP3: 3xP3 promoter, and SV40: SV40 polyadenylation signal sequence. The restriction enzyme sites for the construction of recombinant vectors are indicated with the arrows. (**b**) White-light and fluorescent images of eCFP, eGFP, eYFP and mKate2 silk cocoons produced by silkworm transgenesis via the *piggyBac* transposase method. The fluorescent images are taken with the set of the excitation (λ_{ex}) and emission (λ_{em}) wavelengths, as specified on each photograph. The scale bar is 10 mm. (**c**) Normalized absorption (left) and fluorescence (right) spectra of eCFP (cyan solid line), eGFP (green solid line), eYFP (yellow solid line), and mKate2 (red solid line) transgenic silk. The excitation wavelengths are 415, 470, 470, and 530 nm for eCFP, eGFP, eYFP, and mKate2 silk, respectively.

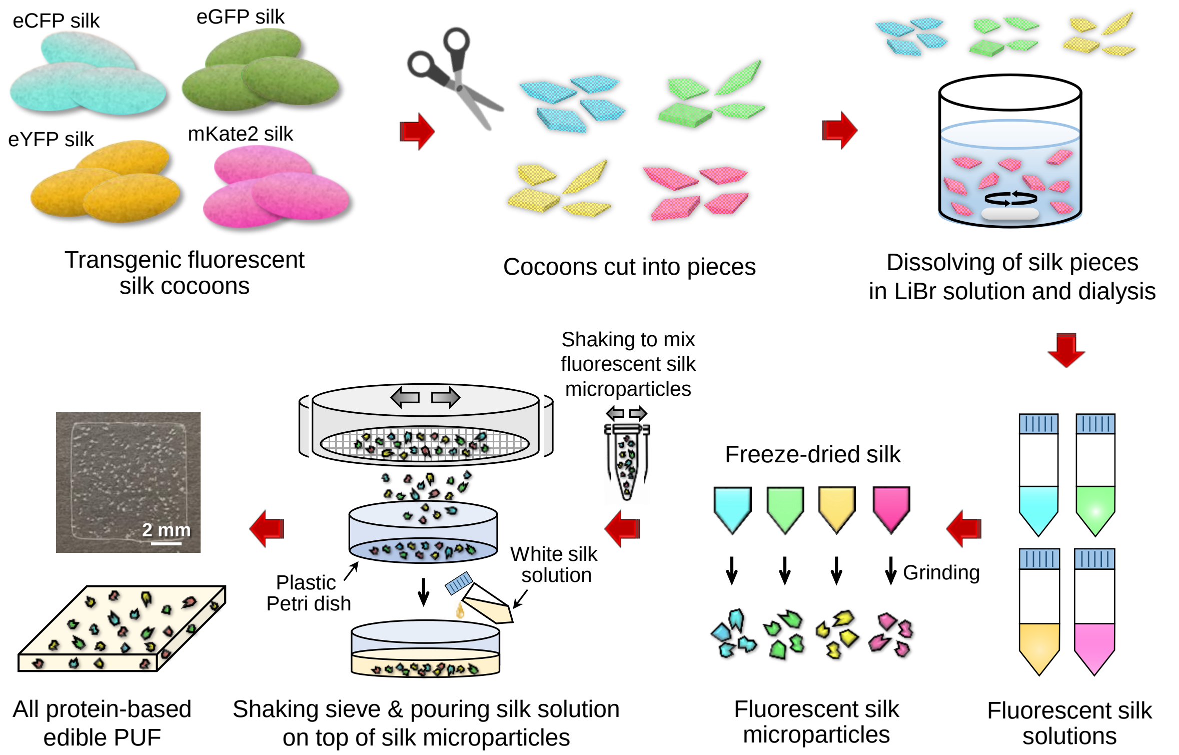

Supplementary Figure 2. Fabrication process of edible PUFs. Schematic diagram of silk protein regeneration and edible PUF device fabrication using fluorescent silk (i.e. eCFP, eGFP, eYFP, and mKate2 silk) and natural white silk.

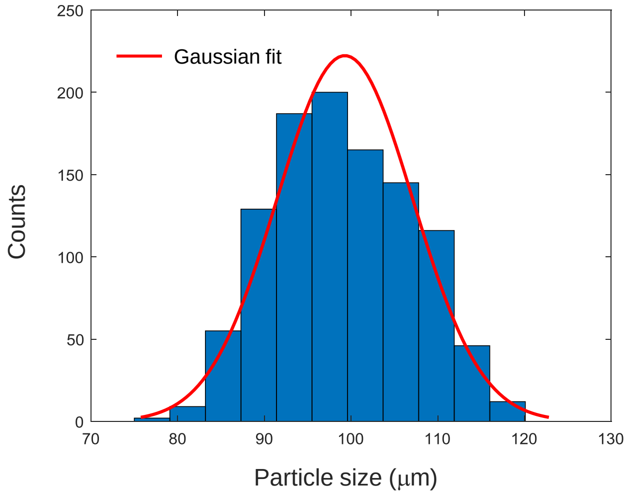

Supplementary Figure 3. Size distribution of the fluorescent silk microparticles. The particle sizes are characterized from SEM images. A Gaussian fit returns a mean of 99.3 μm and a standard deviation (SD) of 7.9 μm. Source data are provided as a Source Data file.

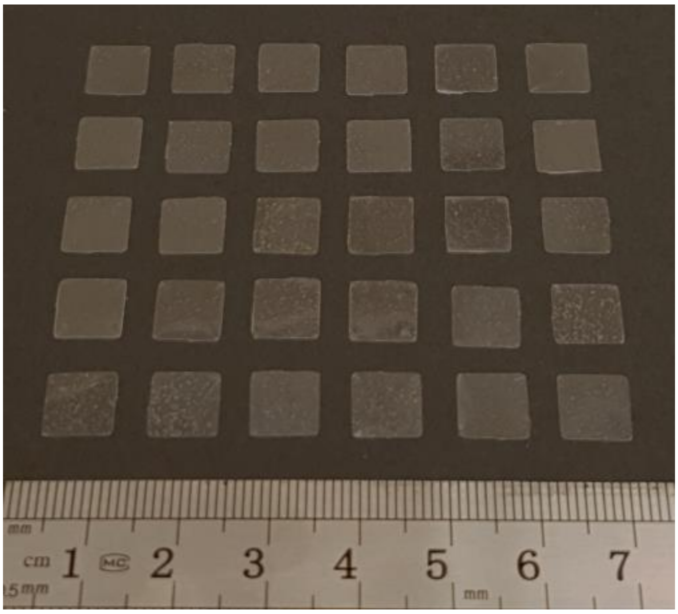

Supplementary Figure 4. Photograph of edible PUFs. 30 different edible PUF devices are used to characterize the overall PUF performance. Each size is 7 × 7 mm^2.

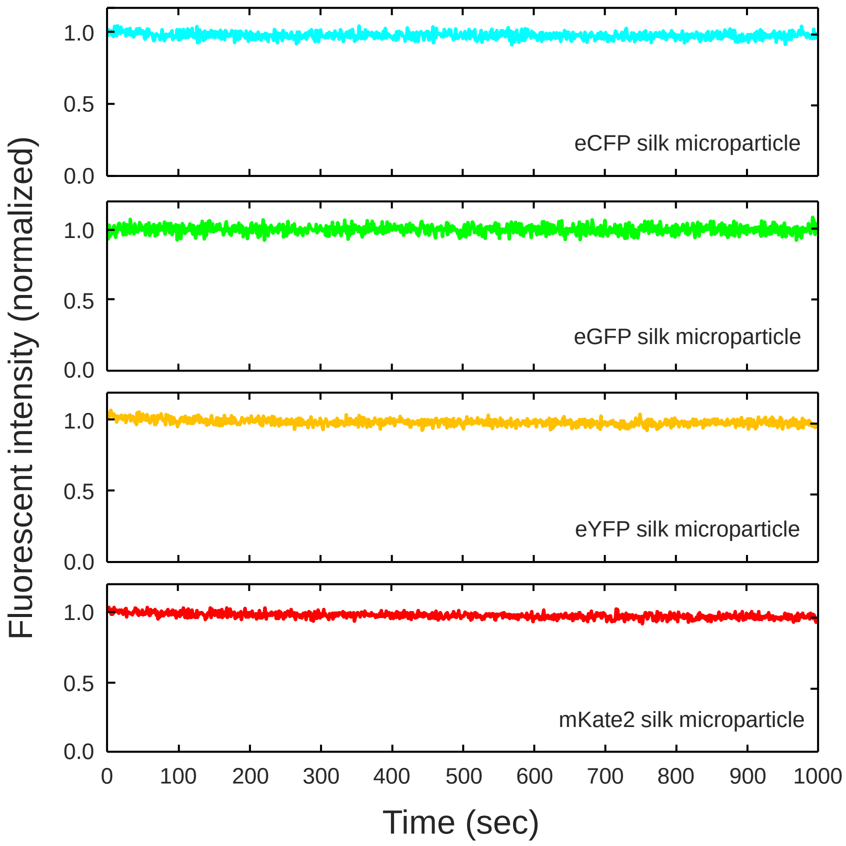

Supplementary Figure 5. Temporal photostability of eCFP, eGFP, eYFP, and mKate2 silk. Fluorescent microparticles are under excitation of 440 nm for eCFP, eGFP, and eYFP silk and 514 nm for mKate2 silk. The emission intensity is measured through a 458-nm longpass filter for eCFP, eGFP, and eYFP silk and a 550-nm longpass filter for mKate2 silk over 1000 seconds.

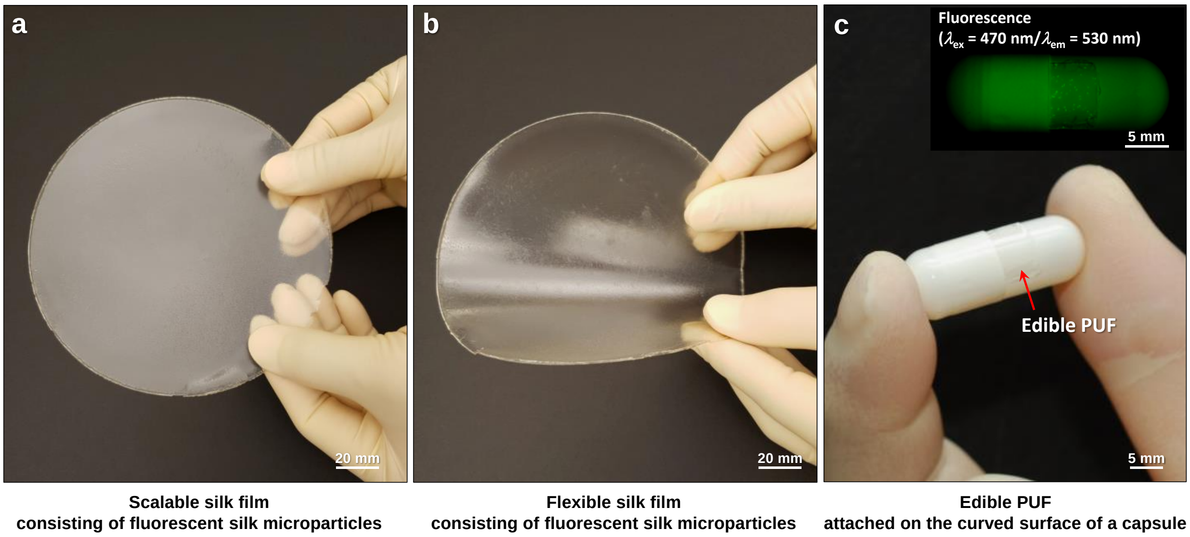

Supplementary Figure 6. Photographs of silk films and feasibility of edible PUFs. Scalability (**a**) and flexibility (**b**) of the proposed edible PUFs for mass production and easy integration with medicines. (**c**) The edible PUF device is attached on the curved surface of a 000-size gelatin capsule that is approved by FDA. As an edible adhesive glue, we used FONDX Edible Glue-Clear (Item code: AMEG2oz; FONDX America Corp., Northridge, CA, USA) to attach the edible PUF to the capsule. Inset: the fluorescent image of the edible PUF is obtained by an Android smartphone (Samsung Galaxy Note 9).

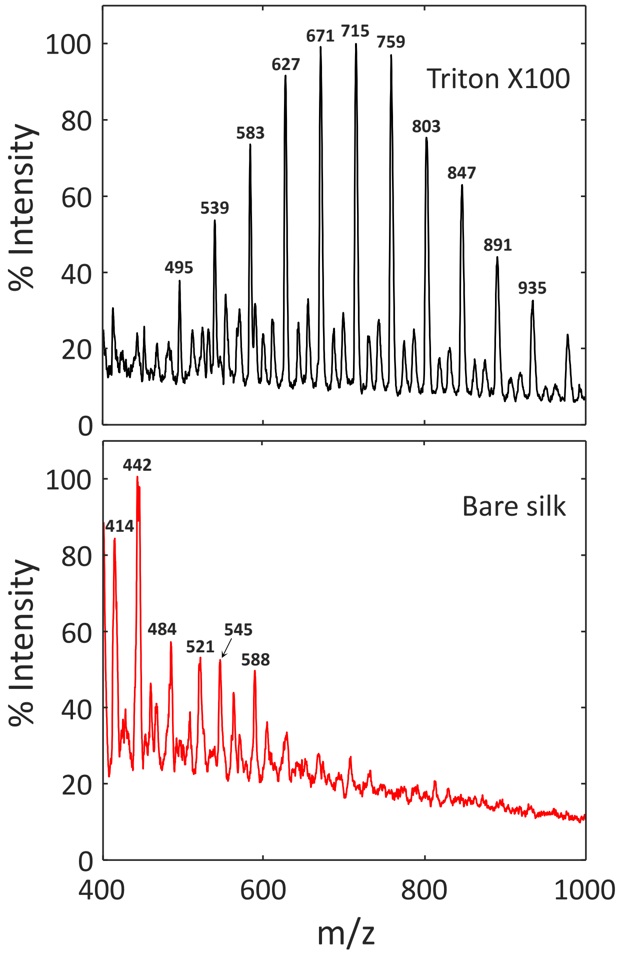

Supplementary Figure 7. MALDI-MS results of regenerated silk. Triton X100 and bare silk are dissolved in deionized water. The peaks of Triton X100 are not observed in the final silk solution, supporting the idea that there is no trace of Triton X100 after the silk regeneration process.

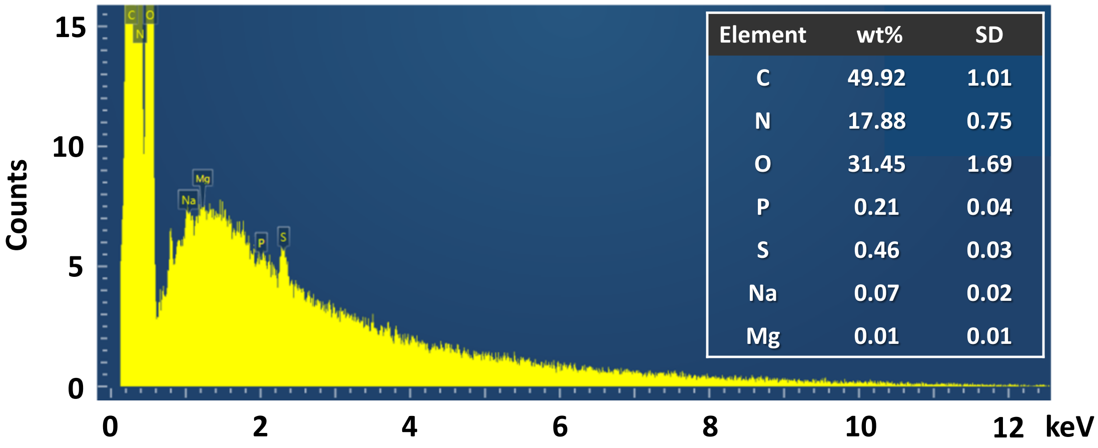

Element	wt%	SD
C	49.92	1.01
N	17.88	0.75
O	31.45	1.69
P	0.21	0.04
S	0.46	0.03
Na	0.07	0.02
Mg	0.01	0.01

Supplementary Figure 8. EDX analysis of edible PUFs. As silk is the main material, the edible PUF devices contain the dominant three elements of carbon (C), nitrogen (N), and oxygen (O), including other minor elements of phosphorus (P), sulfur (S), sodium (Na), and magnesium (Mg). Importantly, a trace of bromine (Br; K_{α} 11.907, L_{α} 1.480) from lithium bromide (LiBr), which is used to dissolve silk cocoons as a special type of salt, is not detected.

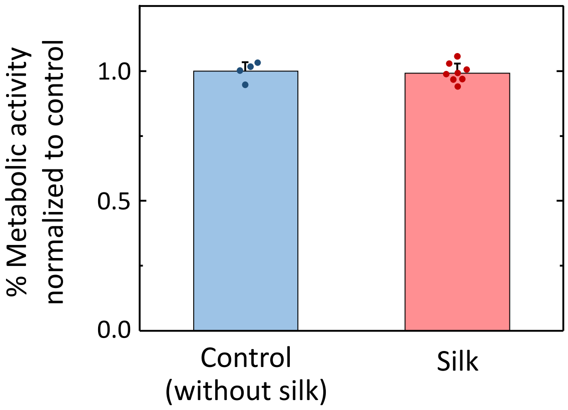

Supplementary Figure 9. General cytotoxicity test of edible PUFs. *In vitro* MTT tests show that the metabolic activity of the silk products is not different from that of the controls (p-value of a two-tailed t-test = 0.81). The error bar is a standard deviation. Source data are provided as a Source Data file.

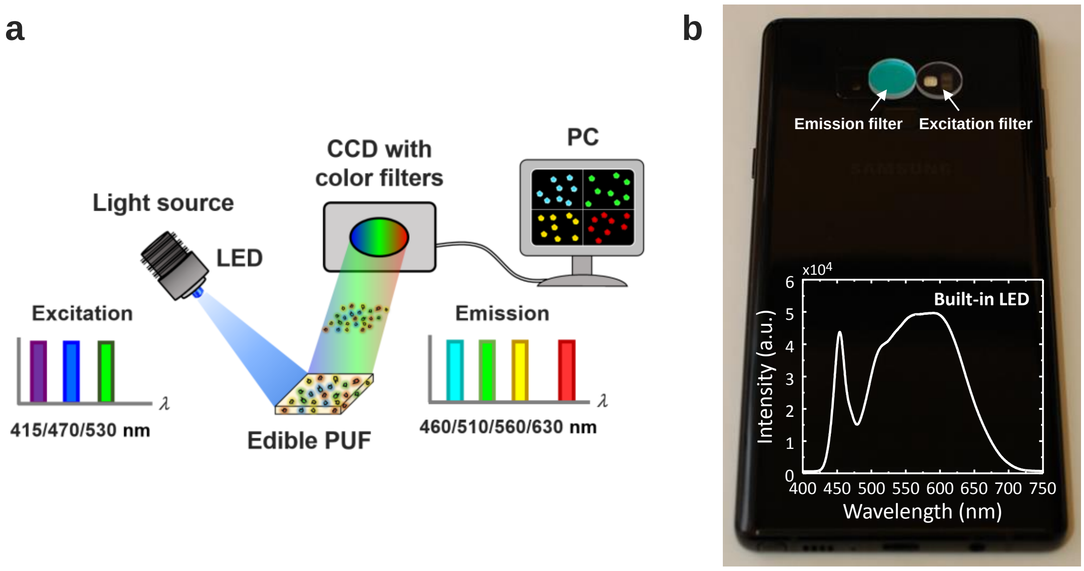

Supplementary Figure 10. Imaging setups for reading PUFs. (**a**) Schematic diagram of the customized imaging setup to obtain fluorescent images of edible PUF devices. As easily accessible common light sources for optical excitation, ultraviolet, blue, and green light-emitting diodes (LEDs) at central wavelengths of 415, 470, and 530 nm are used. Fluorescent images are acquired by a charge-coupled device (CCD) camera through a liquid crystal tunable filter with emission wavelengths of 460, 510, 560, and 630 nm with acquisition times of 60, 5, 40, and 30 sec, respectively. Different combination sets of the excitation and emission wavelengths serve as challenges to realize the parametric support of challenge-response pairs in edible PUFs. (**b**) Photograph of the smartphone used for a feasibility test. An Android smartphone (Samsung Galaxy Note 9) is used as a reader. Inset: the spectrum of the flashlight LED embedded in the smartphone. This built-in LED serves as an excitation light source. A shortpass filter (cutoff $\lambda = 450$ nm) and a bandpass filter ($\lambda = 530$ nm $\pm$ 10 nm) are placed in front of the flashlight LED. The optical intensity is measured to be 0.3 and 0.8 $\mu W\ mm^{-2}$ at $\lambda = 440$ nm and $\lambda = 530$ nm on the surface of PUFs, respectively. The fluorescent images of the PUFs are acquired using the smartphone camera via longpass filters (cutoff $\lambda = 500$ nm and cutoff $\lambda = 600$ nm) with an acquisition time of 1 and 10 seconds, respectively. Given some high-end smartphones already have multiple light sources (e.g. flashlight LED), we envision that multiple different colored LEDs can easily be embedded into a smartphone as manufacturers realize a variety of direct applications, including the proposed on-dose authentication.

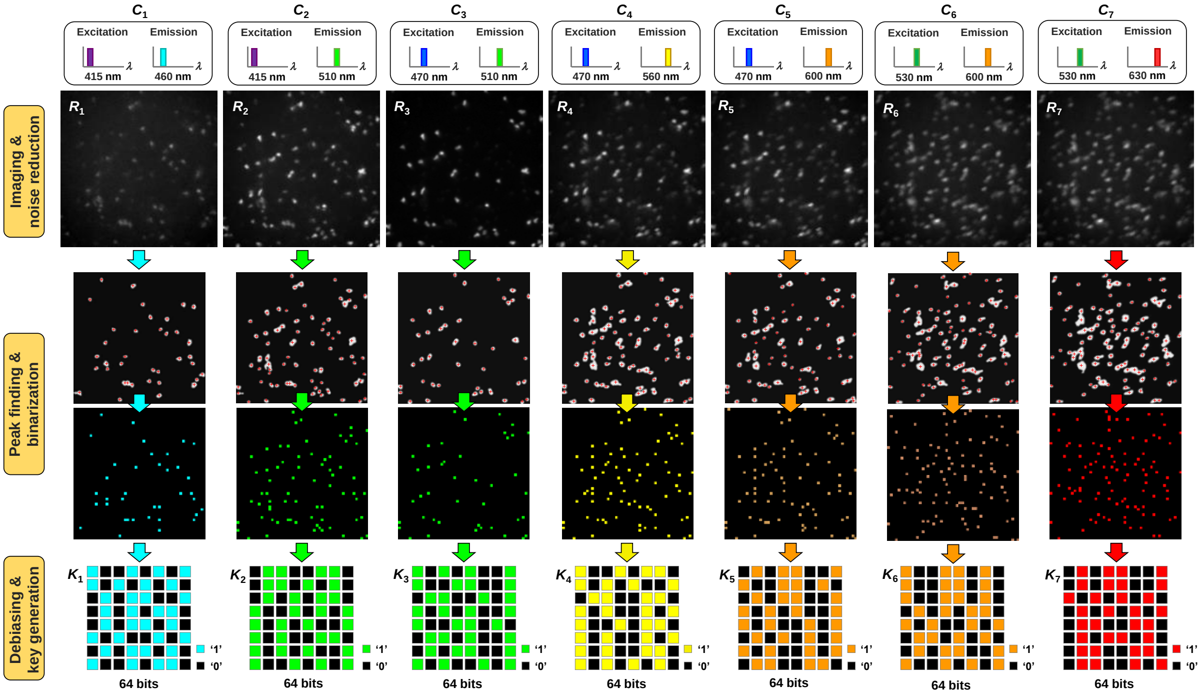

Supplementary Figure 11. Extended number of challenge-response pairs for a stronger PUF. The extractor consists of the same elements of noise reduction, peak position finding, binarization, and debiasing. Seven challenge-response pairs create a total of 448-bit security key with an enhanced nominal encoding capacity of 2^{448} (= 7.2684×10^{134}).

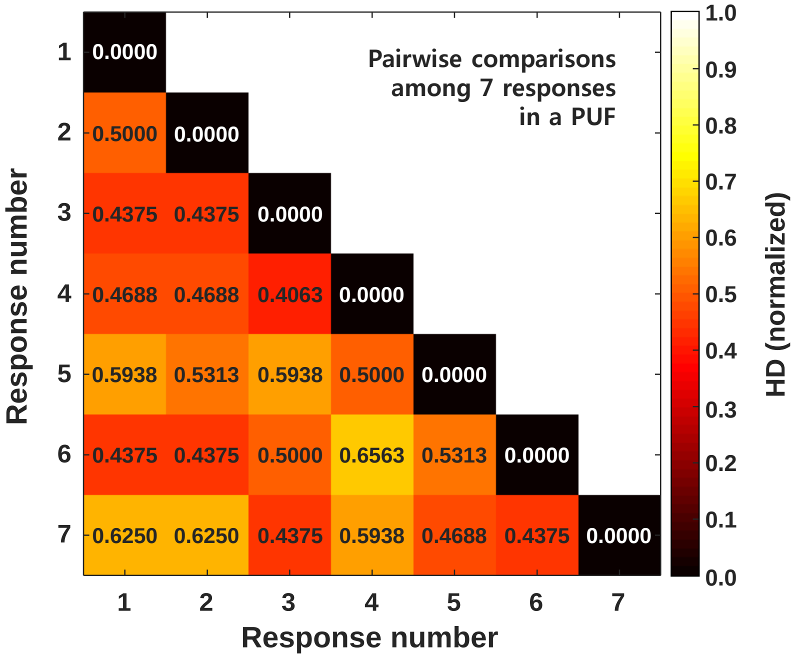

Supplementary Figure 12. Pairwise comparisons among digitized keys from seven different responses in a single PUF. A mean of HDs in the off-diagonal areas is 0.5089 with a SD of 0.0766, supporting minimum correlations among different security keys from each response. In other words, the security key from each output response is unique and distinct.

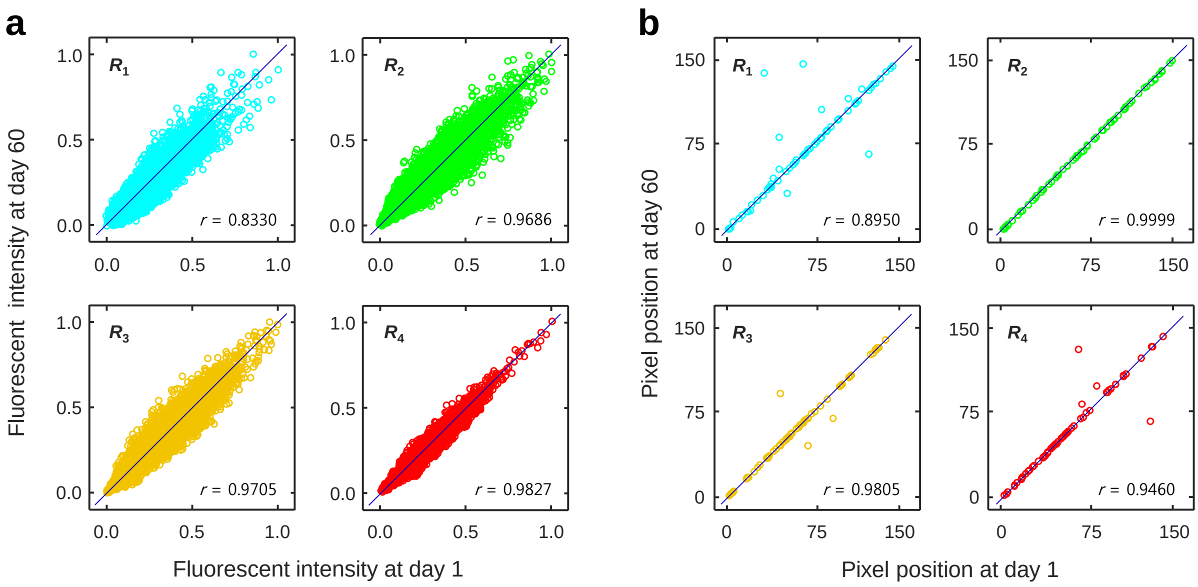

Supplementary Figure 13. Long-term reliability and stability of edible PUFs. (**a**) Scatterplots of the normalized fluorescent intensity of four output responses acquired 60 days apart using the same corresponding challenges. (**b**) Scatterplots of pixel positions (first 32 peaks in the binarized images) of four responses acquired 60 days apart using the same corresponding challenges. *r* is the correlation coefficient. The highly linear relationships support the long-term reliability and stability of the edible PUFs. Source data are provided as a Source Data file.

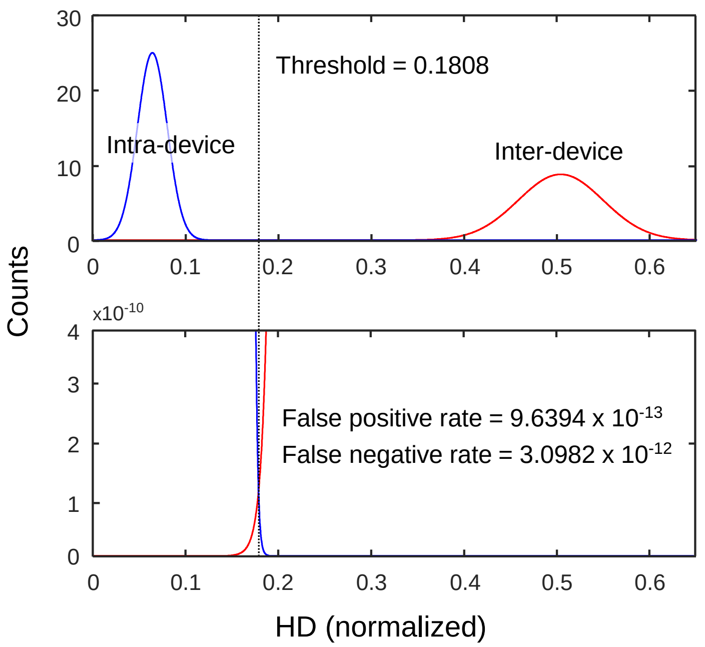

Supplementary Figure 14. Intra-device (readout reproducibility) and inter-device (device uniqueness) variabilities. The resulting false positive and false negative rates with a cut-off threshold of 0.1808 are 9.6394×10^{-13} and 3.0982×10^{-12}, respectively.

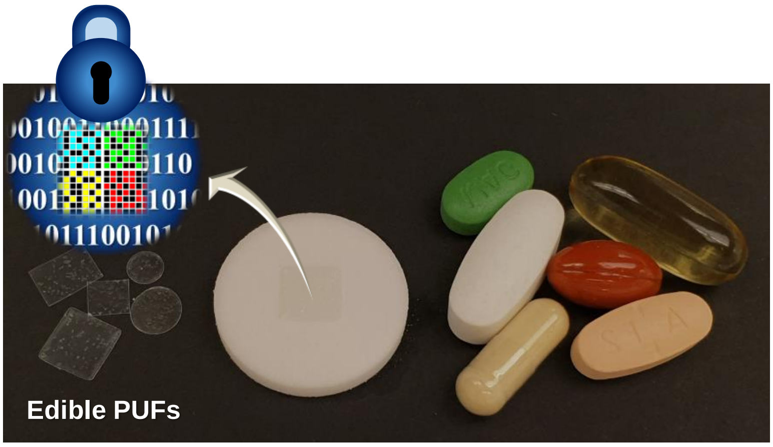

Supplementary Figure 15. Photograph of edible PUFs for anti-counterfeiting of medicines. An edible PUF can directly be attached on the surface of solid medicines for on-dose (or in-dose) authentication.

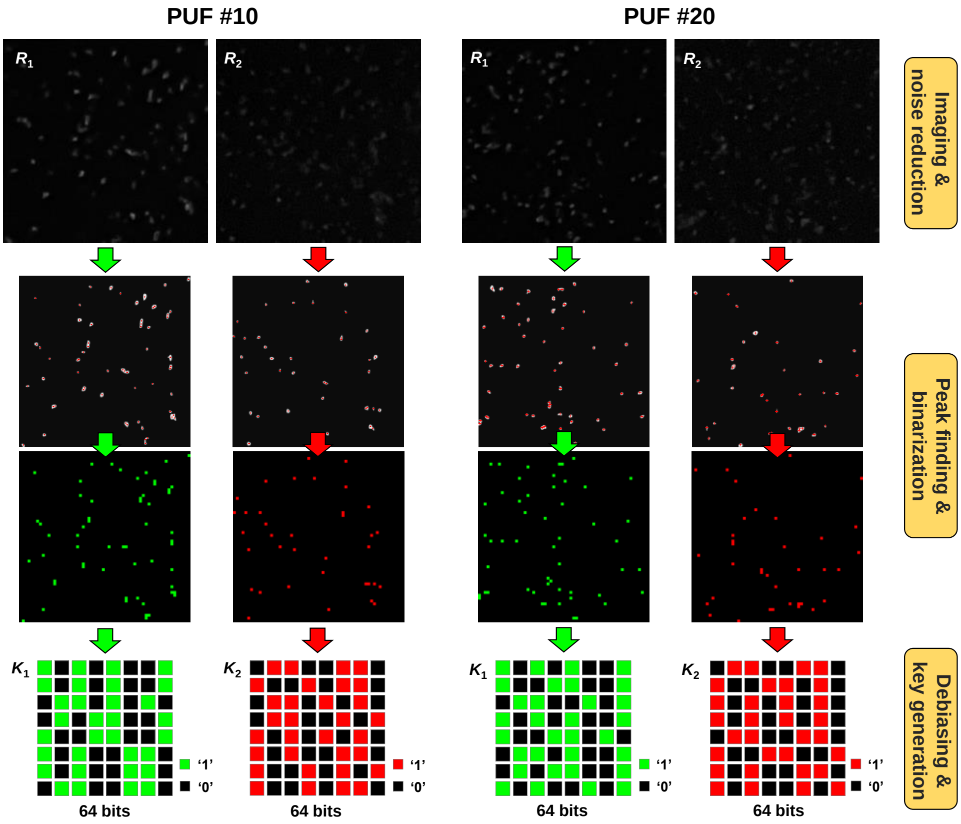

Supplementary Figure 16. Feasibility of using a smartphone as a reader. Representative digitized keys from two different PUF devices using a smartphone (Samsung Galaxy Note 9). In the customized MATLAB codes for image processing and digital key extraction, RGB images are converted to grayscale by the 'rgb2gray' function and are normalized by the maximum fluorescent intensity. Other elements are the same in the extractor used for the customized imaging setup. As an example, the calculated inter-device HD between PUF #10 and PUF #20 is 0.4375. Indeed, the built-in flashlight LED and camera in a smartphone can easily be used as an excitation light source and an imager to generate digitized keys.

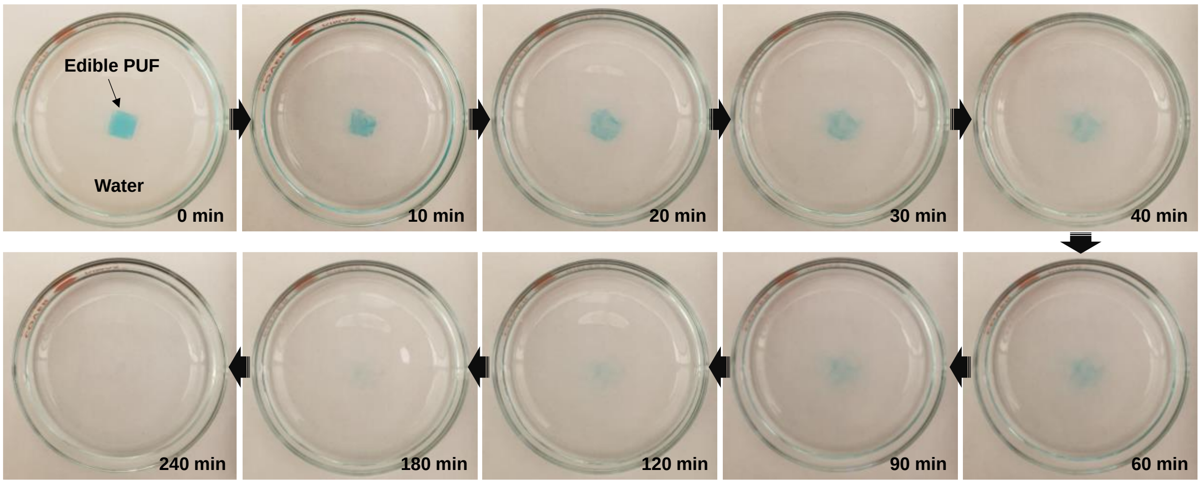

Supplementary Figure 17. Solubility of edible PUFs composed of silk proteins and fluorescent proteins. The natural silk fibroin film embedded with eCFP, eGFP, eYFP, and mKate2 silk microparticles (loaded with methylene blue for easy visualization purpose) in deionized water is completely dissolved in 240 minutes at room temperature.

Supplementary Tables

Supplementary Table 1. Optical properties of fluorescent proteins genetically hybridized with silk

Fluorescent protein	Excitation maximum (nm)	Emission maximum (nm)	Extinction coefficient (M^{-1} cm^{-1})	Quantum yield (%)
eCFP	434	477	32,500	40
eGFP	489	509	55,000	60
eYFP	514	527	84,000	61
mKate2	588	633	62,500	40

Supplementary Table 2. Brief characteristic descriptions of the NIST statistical tests

**Test name**	***n***	***M or m***	**# of sub-tests**	**Defect detected**
Frequency	128	-	1	Proportion of '0's and '1's for the entire sequence, assessing the closeness to 1/2.
Block frequency	128	20	1	Proportion of '0's and '1's within *m*-bit blocks (*m* is the length in bits of each block).
Cumulative sums	128	-	2	Maximal excursion of a random walk, determining the cumulative sum of the partial sequences.
Runs	128	-	1	Number of runs (uninterrupted sequence of identical bits) and the relative oscillation, using the number of runs of '0's and '1's of various lengths.
Longest run of ones	128	8	1	Length of the longest run of '1's within the tested sequence, compared with that from a random sequence.
Approximate entropy	128	2	1	Frequency of overlapping patterns across the entire sequence, using the frequency of overlapping blocks of two consecutive/adjacent lengths. Similar to serial test.
Serial	128	4	2	Frequency of overlapping patterns across the entire sequence, checking the number of occurrences of the 2^m *m*-bit overlapping patterns. Similar to approximate entropy.

n is the size of the sequence length. *M* or *m* is the length in bits of each block.

Supplementary Methods

Construction of plasmid vector DNA for silkworm transgenesis

Using germline transformation (i.e. *piggyBac* transposon), the transition vectors pBac-3xP3-DsRed2-pFibH-(eCFP, eGFP, or eYFP) and pBac-3xP3-eGFP-pFibH-mKate2 were constructed as the *piggyBac*-derived vector and the injected the vector DNA with a helper vector into pre-blastoderm embryos (**Supplementary Figure 1a**)[1-6]. For construction of plasmids, DsRed2 (eGFP for mKate2) cDNA as a marker was amplified by polymerase chain reaction (PCR) using specific primers with *Nhe*I/*Afl*II sites from pDsRed2-C1 (*Nhe*I-DsRed2-F: 5′-GCTAGCATGGCCTCCTCCGAGAAC-3′ and DsRed2-*Afl*II-R: 5′-CTTAAGCTACAGGAACAGGTGGTGGCG-3'; Clontech, Mountain View, CA, USA) and then cloned into the pGEM-T Easy Vector System (Promega, Co.), designated as pGEMT-DsRed2. DsRed2 gene was excised from pGEM-DsRed2 digested with restriction enzymes of *Nhe*I/*Afl*II and replaced with eGFP gene from pBac-3xP3-eGFP to form pBac-3xP3-DsRed2. To obtain fibroin promoter, a DNA fragment containing the promoter domain (1,124 bp) and N-terminal region (1,430 bp) with the intron (972 bp) of the fibroin H gene (GenBank Accession No. AF226688, nt. 61,312-63,870) was amplified by PCR using the genomic DNA from *Bombyx mori* and primers (pFibHN-F: 5′- GGCGCGCCGTGCGTGATCAGGAAAAAT-3′ and pFibHN-R: 5′-TGCACCGACTGCAGCACTA GTGCTGAA-3′), subsequently cloned into pGEM-T Easy Vector System. The resultant DNA fragment was named as pGEMT-pFibH-NTR. The DNA fragment containing 180 bp of the 3′ terminal sequence of the H-chain gen open reading frame and 300 bp of the 3′ region of the fibroin H gen (GenBank Accession No. AF226688, nt. 79,021-80,009) was amplified by PCR using genomic DNA from *Bombyx mori* and primers (pFibHC-F: 5′-AGCGTCAGTTACG GAGCTGGCAGGGGA-3′ and pFibHC-R: 5′-TATAGTATTCTTAGTTGAG AAGGCATA-3′) and then was cloned into pGEM-T Easy Vector System. This fragment is designated as pGEMT-CTR. The fragments were prepared by digesting pGEMT-pFibH-NTR with *Asc*I/*Sal*I and pGEMT-CTR with *Sal*I/*Sac*I, respectively. These two fragments were cloned with pBluescriptII SK(-) vector (Stratagene) digested with *Apa*I/*Sal*I, resulting in pFibHNC-null. Fluorescent genes (eCFP, eGFP, eYFP, and mKate2) were synthesized and purchased from the BIONEER Corporation (South Korea). The N- and C-terminals had the *Not*I and *Sbf*I restriction sites, respectively. The fluorescent genes were digested with *Not*I/*Sbf*I and subcloned into a pFibHNC-null, resulting in pFibHNC-eCFP, pFibHNC-eGFP, pFibHNC-eYFP, and pFibHNC-mKate2, respectively. The resultant vector was named as pBac-3xP3-eCFP-FibH, pBac-3xP3-DsRed2-FibH-eGFP, pBac-3xP3-DsRed2-pFibH-eYFP, and pBac-3xP3-eGFP-FibH-mKate2, respectively.

Sericin removal (degumming)

Sericin of transgenic silk cocoons was removed with minimizing heat-induced denaturation of fluorescent proteins (i.e. eCFP, eGFP, eYFP, and mKate2)[7,8]. The silk cocoons were treated several times with a mixture solution of Na_2CO_3 (0.2%) and Triton X100 (0.1%) at temperatures lower than 50 °C, subsequently rinsed with warm deionized water (≈ 35 °C) several times. During the degumming process, the low pressure (620 mmHg) was additionally treated to completely remove the sericin. After degumming, the sericin-removed cocoons were dried under dark ambient conditions. For white silk, we used a conventional degumming method under a boiling process[9,10]. The result from mass spectroscopy showed that there were no residual components of Triton X100 (**Supplementary Methods and Supplementary Figure 7**). It should be noted that Na_2CO_3 (also known as soda crystals) is an inactive ingredient for drug products approved by FDA[11]. In addition, it is well known that natural silk also contains the elements of carbon (C), oxygen (O), and sodium (Na)[5,12].

Scanning electron and confocal scanning microscopy

We obtained scanning electron microscopy (SEM) images and energy dispersive X-ray (EDX) data using FEI Quanta 3D FEG (Oregon, USA) at 10 kV for SEM and 20 kV for EDX, respectively. For each fluorescent silk, fluorescent images of the microparticles were collected using laser scanning confocal microscopy with a Zeiss LSM 880 confocal microscope (Carl Zeiss GmbH, Jena, Germany)

with a 10× lens. The *x* and *y* pixel length was 2048. We used 405-nm, 458-nm, 514-nm, and 561-nm lasers as excitation sources and set detection bandpass emission filters of 460 ± 10, 510 ± 10, 560 ± 10, and 630 ± 10 nm for eCFP, eGFP, eYFP, and mKate2 silk microparticles, respectively. The typical parameters of confocal microscopy were set to be confocal aperture (pinhole) size of 84 μm and scan speed (pixel dwell time) = 2 μs pixel^{-1}, respectively.

Photoluminescent properties of fluorescent silk microparticles
Absorption and fluorescent spectra of individual fluorescent silk microparticles were measured under optical excitation of 415 nm for eCFP silk, 470 nm for eGFP silk and eYFP silk, and 530 nm for mKate2 silk, by using a fiber bundle-coupled spectrometer (VS140 VIS-NIR; Horiba Jobin Yvon Inc., Edison, NJ, USA) and a microscope coupled with an imaging spectrograph (IsoPlane-320, Princeton Instruments, Inc., Trenton, NJ, USA). Absorption (= 1 – reflectance – transmittance) spectra of eCFP, eGFP, eYFP, and mKate2 silk were measured with an integrating sphere. Time traces of the fluorescent emission intensity for eCFP, eGFP, eYFP, and mKate2 silk microparticles were monitored with a 458-nm longpass filter for eCFP, eGFP, eYFP silk and a 550-nm longpass filter for mKate2 silk over 1000 seconds under optical excitation at 440 nm for eCFP, eGFP, eYFP silk and 514 nm for mKate2 silk, respectively.

Mass spectroscopy
We investigated whether a trace of Triton X100 in the silk solution was detectable with matrix-assisted laser desorption/ionization-mass spectrometry (MALDI-MS), using a Voyager-DE PRO mass spectrometer (Applied Biosystems, Foster City, CA, USA). For comparison, a control sample consisting of 10% (v v^{-1}) triton X100 dissolved in deionized water was also prepared. 1 μl of the sample was added to 1 μl of matrix and spotted on the MALDI plate. The matrix consisted of 10 mg ml-1 a-cyano-4-hydroxycinnamic acid in 50:50 acetonitrile: 0.1 % trifluoroacetic acid in deionized water. Spectra were obtained in the linear mode with delayed extraction with an accelerating voltage of 25 kV. From MALDI-MS data, the peaks of Triton X100 were not observed in the silk solution, supporting the idea that there was no residue of Triton X100 after the silk regeneration process (**Supplementary Figure 7**).

Cytotoxicity (cell viability) assay
We conducted MTT assays *in vitro* to measure the general cytotoxicity (cell viability) of the silk samples. The silk samples were emerged in a PBS solution for 48 hours and the solution was diluted in a ratio of 1:10 in MEM 2% FBS and layered on Baby Hamster Kidney fibroblasts (also known as BHK cells). Cell viability after four days was determined with the MTT assay in triplicate. Eight independent experiments and four controls were performed under the same conditions. The metabolic activity of the silk products was not different from that of the controls (p-value of a two-tailed t-test = 0.81) (**Supplementary Figure 9**).

Cost analysis
The material cost of the edible PUF devices can be estimated to be \$0.01 – \$0.02 per item. This corresponds to 50 – 60 mg of silk fibroin in each PUF. A typical price of 100 pieces of silk cocoons (*Bombyx mori*) is approximately \$9.00 from online stores. In other words, one silk cocoon costs \$0.09. Using three silk cocoons, we prepare for a 10-mL silk fibroin solution (4% w v^{-1}). 4 mL of the silk solution is poured onto a plastic petri dish with a diameter of 35 mm, resulting in a film thickness of 150 μm. Then, seven PUF devices with a size of 7 × 7 mm^2 are punched out. As a result, the cost of one edible PUF is approximately \$0.015 each. This corresponds to 50 – 60 mg of silk fibroin in each PUF, because our silk film weighs 1.0 – 1.2 mg mm^{-2} (one silk cocoon weighs about 300 – 350 mg on average). Even if the small amount of fluorescent silk microparticles is considered, the final cost would be less than \$0.02 each.

Supplementary References

1 Kim, D. et al. Novel fabrication of fluorescent silk utilized in biotechnological and medical applications. *Biomaterials* **70**, 48-56, (2015).
2 Tamura, T. et al. Germline transformation of the silkworm *Bombyx mori* L. using a *piggyBac* transposon-derived vector. *Nature Biotechnology* **18**, 81-84, (2000).
3 Teule, F. et al. Silkworms transformed with chimeric silkworm/spider silk genes spin composite silk fibers with improved mechanical properties. *Proceedings of the National Academy of Sciences of the United States of America* **109**, 923-928, (2012).
4 Iizuka, T. et al. Colored fluorescent silk made by transgenic silkworms. *Advanced Functional Materials* **23**, 5232-5239, (2013).
5 Leem, J. W. et al. Scalable and continuous nanomaterial integration with transgenic fibers for enhanced photoluminescence. *Materials Horizons* **4**, 281-289, (2017).
6 Leem, J. W. et al. Green-light-activated photoreaction via genetic hybridization of far-red fluorescent protein and silk. *Advanced Science* **5**, 1700863, (2018).
7 Nagy, A., Malnasi-Csizmadia, A., Somogyi, B. & Lorinczy, D. Thermal stability of chemically denatured green fluorescent protein (GFP) - A preliminary study. *Thermochimica Acta* **410**, 161-163, (2004).
8 Alkaabi, K. M., Yafea, A. & Ashraf, S. S. Effect of pH on thermal- and chemical-induced denaturation of GFP. *Applied Biochemistry and Biotechnology* **126**, 149-156, (2005).
9 Tao, H., Kaplan, D. L. & Omenetto, F. G. Silk materials - A road to sustainable high technology. *Advanced Materials* **24**, 2824-2837, (2012).
10 Rockwood, D. N., Preda, R. C., Yucel, T., Wang, X. Q., Lovett, M. L. & Kaplan, D. L. Materials fabrication from *Bombyx mori* silk fibroin. *Nature Protocols* **6**, 1612-1631, (2011).
11 FDA, Inactive ingredient search for approved drug products. (Accessed in October, 2019) <https://www.accessdata.fda.gov/scripts/cder/iig/index.cfm>.
12 Tulachan, B. et al. Electricity from the silk cocoon membrane. *Scientific Reports* **4**, 5434 (2014).
